# Supplementary material for: ProteinShader: illustrative rendering of macromolecules
Source: BMC Struct Biol. 2009 Mar 30;9:19. doi: 10.1186/1472-6807-9-19 (PMC2672931; doi:10.1186/1472-6807-9-19)
Supplement: Additional file 1 — ProteinShader program without source code. This compressed file contains the complete ProteinShader program including associated libraries, but no source code. A README.txt file gives an overview of the ProteinShader distribution, and the index.html file in the help subdirectory has directions on getting started with the program as well as a set of tutorials. [file 1472-6807-9-19-S1.zip › ProteinShader-beta-0_9_4-binary/help/api/org/proteinshader/structure/enums/class-use/SideChainEnum.html]

Uses of Class org.proteinshader.structure.enums.SideChainEnum (ProteinShader API)


|  |  |  |
| --- | --- | --- |
| **Overview** \| **Package** \| **Class** \| **Use** \| **Tree** \| **Deprecated** \| **Index** \| **Help** | |  |
| PREV   NEXT | **FRAMES**    **NO FRAMES**     **All Classes** |  |


---


## **Uses of Class org.proteinshader.structure.enums.SideChainEnum**

| Packages that use SideChainEnum | |
| --- | --- |
| **org.proteinshader.gui.components.controlpanel** | Holds the JPanels used in the right-side retractable control panel of the GUI. |
| **org.proteinshader.structure** | Holds the classes that store information from a Protein Data Bank file: Structure, Model, Chain, AminoAcid, Heterogen, Water, Atom, Bond, Helix, BetaStrand, Loop, *etc*. |
| **org.proteinshader.structure.enums** | Holds the enums needed by org.proteinshader.structure and its subpackages. |
| **org.proteinshader.structure.visitor.modifiers** | Holds helper classes for the ModifierVisitor class, which is used to modify Drawable objects (Atoms, Bonds, and Segments). |

Uses of SideChainEnum in org.proteinshader.gui.components.controlpanel

| Methods in org.proteinshader.gui.components.controlpanel with parameters of type SideChainEnum | |
| --- | --- |
| `void` | `CartoonSideChainsPanel.setSideChainStyle(SideChainEnum sideChainStyle)`             Applies the requested side chain style (NONE, SPACE\_FILLING, BALLS\_AND\_STICKS, or STICKS) to whatever item or items are currently selected in the RadioPanel. |

Uses of SideChainEnum in org.proteinshader.structure

| Methods in org.proteinshader.structure that return SideChainEnum | |
| --- | --- |
| `SideChainEnum` | `Segment.getSideChainStyle()`             Gets the side chain style (NONE, SPACE\_FILLING, STICK\_AND\_BALLS, or STICKS). |

| Methods in org.proteinshader.structure with parameters of type SideChainEnum | |
| --- | --- |
| `void` | `Segment.setSideChainStyle(SideChainEnum style, boolean alphaCarbon)`             Sets the side chain style (NONE, SPACE\_FILLING, STICK\_AND\_BALLS, or STICKS) and determines whether the alpha-carbon should be shown. |

Uses of SideChainEnum in org.proteinshader.structure.enums

| Methods in org.proteinshader.structure.enums that return SideChainEnum | |
| --- | --- |
| `static SideChainEnum` | `SideChainEnum.getDefaultSideChainType()`             Returns the default side chain type, which is NONE. |
| `static SideChainEnum` | `SideChainEnum.valueOf(String name)`             Returns the enum constant of this type with the specified name. |
| `static SideChainEnum` | `SideChainEnum.valueOfMenuName(String menuName)`             Returns the SideChainEnum with the same menu name as the String given as an argument. |
| `static SideChainEnum[]` | `SideChainEnum.values()`             Returns an array containing the constants of this enum type, in the order they're declared. |

Uses of SideChainEnum in org.proteinshader.structure.visitor.modifiers

| Methods in org.proteinshader.structure.visitor.modifiers with parameters of type SideChainEnum | |
| --- | --- |
| `void` | `SegmentModifier.setSideChainStyle(SideChainEnum sideChainStyle, boolean displayAlphaCarbon)`             Stores a memory that the side chain display needs to be set. |

---


|  |  |  |
| --- | --- | --- |
| **Overview** \| **Package** \| **Class** \| **Use** \| **Tree** \| **Deprecated** \| **Index** \| **Help** | |  |
| PREV   NEXT | **FRAMES**    **NO FRAMES**     **All Classes** |  |


---

# *Copyright © 2007-2008*
